# A multiresolution framework to characterize single-cell state landscapes

**DOI:** 10.1038/s41467-020-18416-6

**Published:** 2020-10-26

**Authors:** Shahin Mohammadi, Jose Davila-Velderrain, Manolis Kellis

**Affiliations:** 1 MIT Computer Science and Artificial Intelligence Laboratory, Cambridge, MA 02139 USA; 2 Broad Institute of MIT and Harvard, Cambridge, MA 02142 USA

**Keywords:** Machine learning, Software, Transcriptomics

## Abstract

Dissecting the cellular heterogeneity embedded in single-cell transcriptomic data is challenging. Although many methods and approaches exist, identifying cell states and their underlying topology is still a major challenge. Here, we introduce the concept of multiresolution cell-state decomposition as a practical approach to simultaneously capture both fine- and coarse-grain patterns of variability. We implement this concept in ACTIONet, a comprehensive framework that combines archetypal analysis and manifold learning to provide a ready-to-use analytical approach for multiresolution single-cell state characterization. ACTIONet provides a robust, reproducible, and highly interpretable single-cell analysis platform that couples dominant pattern discovery with a corresponding structural representation of the cell state landscape. Using multiple synthetic and real data sets, we demonstrate ACTIONet’s superior performance relative to existing alternatives. We use ACTIONet to integrate and annotate cells across three human cortex data sets. Through integrative comparative analysis, we define a consensus vocabulary and a consistent set of gene signatures discriminating against the transcriptomic cell types and subtypes of the human prefrontal cortex.

## Abstract (web summary)

Dissecting the cellular heterogeneity embedded in single-cell transcriptomic data is challenging. Here, the authors introduce the concept of multiresolution cell-state decomposition as a practical approach to simultaneously capture both fine- and coarse-grain patterns of variability.

## Introduction

Single-cell genomic technologies are revolutionizing the way tissues and cell populations are experimentally interrogated. Single-cell approaches are rapidly replacing conventional tissue-level profiling techniques, generating massive data sets in the form of cell, tissue, or organismal atlases^1–4^. Along with technological developments, single-cell biology brings new conceptual challenges. Foremost among the latter is the definition of cell identity itself, and in particular, our interpretations of a cell type and its associated dynamical states^5–7^. Technical and conceptual progress in such matters largely depends on the availability of flexible computational frameworks able to efficiently extract dominant transcriptional patterns that discriminate not only distinct cell populations, but also those patterns that might be shared across cell types.

In the context of single-cell transcriptomic analysis, and particularly within frameworks aiming at characterizing the structure of underlying cell states, matrix decomposition techniques are among the most popular approaches^8^. There are multiple different techniques, yet, in essence, such methods aim to decompose a transcriptional profile into a small number of components or patterns that are presumed to optimally represent the transcriptional variability within the data set. As part of the decomposition, the relative contribution of these patterns to the transcriptome of each cell is estimated, along with the relative contribution of genes discriminating each pattern from the others. Principal component analysis (PCA), independent component analysis (ICA), and non-negative matrix factorization (NMF) are among the methods more commonly used^9–14^.

Archetypal analysis (AA)^15^ is a decomposition technique that is much less frequently used, but that nonetheless offers several advantages. First and foremost, AA, by design, produces a more interpretable decomposition, as the underlying learned patterns are expressed as combinations of a parsimonious set of input data points^16^. In addition, unlike NMF and ICA decompositions, AA does not suffer from rotational ambiguity^17^. To demonstrate the benefit of this approach for single-cell transcriptomic analysis, we recently developed the AA for cell-type identification (ACTION) method^18^. ACTION extends AA by coupling it with separable NMF, a variant of NMF that is guaranteed to find the unique global optimum solution^19^. This coupling ensures the reproducibility of AA solutions while improving convergence and efficiency. In the context of single-cell transcriptomics, we showed that ACTION learns interpretable cell states with superior performance relative to more conventional methods^18^.

Despite the power and broad applicability of matrix decomposition methods, they suffer from several technical limitations. A priori selection of the number of underlying dominant patterns to be learned is required but not trivial to determine, nor is the certainty of robustness and reproducibility of the patterns. To simultaneously address these limitations and provide a systematic way to learn cell-type discriminatory and shared dominant transcriptional patterns, here we present a computational framework built upon the concept of multiresolution cell-state decomposition, an approach that systematically prunes and unifies informative patterns identified at different levels of resolution. The outcome of this method is a nonredundant, multiresolution set of cell states whose relative contribution optimally represents the heterogeneity of the entire single-cell transcriptomic data set. To operationalize this idea, we combine the complementary benefits of AA, network theory, and manifold learning for single-cell analysis. We show that this viewpoint for data analysis enables the identification, operationalization, and interpretation of both transcriptional identity and activity states (e.g., cells of different types sharing a functionally similar activation state), a feature not readily available in state-of-the-art clustering-based methodologies. Operationally, this is achieved by introducing a multilevel decomposition and a multiresolution cell-state discovery approach that circumvents technical problems associated with transcriptomic decomposition, while accounting for a potential intrinsic biological property inherent to single-cell data: the existence of multiple meaningful levels of resolution that prohibit the specification of a single optimal number of clusters/components to partition the data.

We implement this framework in the ACTIONet computational environment (https://github.com/shmohammadi86/ACTIONet). Using various data sets; we show how ACTIONet can perform a wide range of single-cell analysis tasks, including dominant pattern and cell-state discovery, network construction and visualization, cell annotation and interpretation, and data integration. Using extensive comparative analyses, we demonstrate ACTIONet’s superior performance to that of existing methods in each methodological step.

## Results

### Overview

We introduce ACTIONet, a comprehensive computational framework for single-cell analysis. Unlike state-of-the-art clustering- or decomposition-based methods, ACTIONet implements the concept of multiresolution decomposition, an approach that enables data-driven identification of both coarse- and fine-grained transcriptional patterns defining discrete and continuous cell states. We show that such underlying state patterns are more interpretable than those found by more conventional alternatives. Multiresolution decomposition is achieved by defining a lower dimensional representation of single-cell transcriptomes that synthesizes information from multiple levels of resolution into a unique pattern-based cell-state representation. ACTIONet uses a modified version of archetypal analysis (ACTION) to learn dominant transcriptional patterns representative of transcriptional cell types and states, and manifold learning to construct a structural representation of the cell-state space. Together, this approach produces a useful lower dimensional embedding for cells and patterns that enables exploration, visualization, and computation. In what follows we individually introduce the components that constitute the core concept of multiresolution cell-state decomposition, and that build up ACTIONet framework. At each step, we demonstrate ACTIONet enhanced performance relative to comparable alternative methods.

### Interpretable matrix decomposition

One of the main goals of single-cell analysis is to identify groups of cells that share transcriptional signatures. To that end, the most common approach is to apply clustering algorithms over a nearest-neighbor graph connecting cells, the latter usually constructed from the PCA decomposition of a subset of highly variable genes (HVGs)^20^. Alternatively, one might aim to identify patterns that describe the transcriptome of cellular subpopulations in the data through matrix factorization, and to quantify the contribution of such patterns to the transcriptome of each cell. Factors that contribute highly and uniquely to a subset of cells are expected to map well onto independently resolved clusters. ACTIONet fits in this latter approach type, having a decomposition technique (ACTION) as a core component. Regardless of the approach (clustering or decomposition), the aim is to recover true signals corresponding to bonafide biological cell types or states. What constitutes a true signal in real data sets is generally not known or hard to quantify. Therefore, we first use artificially designed (synthetic) data to demonstrate the power of ACTIONet’s decomposition component to identify different types of real signals.

**Fig. 1:**
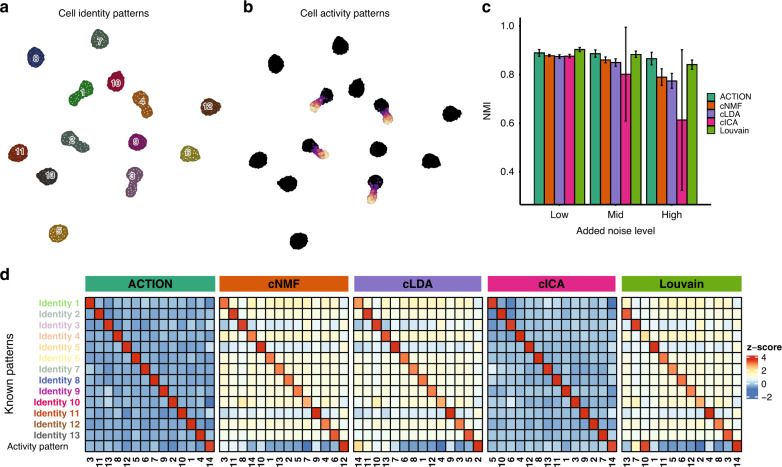
ACTION decomposition robustness and interpretability. **a** Performance of ACTIONet framework in recovering both identity and activity cell patterns. Cells of the same identity but with variable activity pattern form a gradient radiating from the core. **b** Performance of ACTIONet in identifying cell identities across 20 instances at three different noise levels (evaluating matrix H). **c** Performance of ACTIONet in recovering pure and interpretable identity and activity patterns or gene expression profiles (evaluating matrix W). *n* = 20 independent samples over each of *n* = 3 independent noise levels. Data are presented as mean values ±SD. **d** Consistency of learned (columns) and predicted (rows) patterns for the different methods tested, considering the case of 14 known patterns.

The most intuitive type of transcriptional signature to be discovered is a pattern directly consistent with the biological concept of cell type, that is, a discretely and collectively distinct group of cells that share a signature with each other but not with cells outside the group. We refer to this type of signal as cell identity patterns. The second type is the one in which cells that have different identities, also share a common pattern. We refer to this second type of signal as cell activity patterns (Fig. 1a, b). To demonstrate ACTIONet’s power to recover both identity and activity patterns we leveraged a comprehensive synthetic data set that by design includes both pattern types. The data set was generated previously in ref. ^12^. We compared ACTIONet’s performance with that of three popular matrix decomposition techniques: NMF^9^, latent Dirichlet allocation (LDA)^21^, and ICA^22^. All of these methods have been previously applied to identify cell identity patterns^13,23–25^. Given the stochastic nature of these algorithms, they very often produce variable results across different runs. As a way to address output uncertainty, a consensus learning approach has recently been proposed and applied to all three methods, resulting in consensus-variants named cNMF, cLDA, and cICA^12^. In brief, using this modification, a consensus is achieved by performing matrix decomposition multiple times and clustering the resulting factors, after outlier removal. Here, we used the improved, consensus versions to benchmark ACTIONet’s performance. We additionally included the Lovain graph-based clustering algorithm, defining state patterns as cluster averages.

We applied all algorithms to data sets generated using a fixed number of known underlying patterns, and with multiple levels of added noise. We found in all cases that ACTIONet is able to simultaneously recover all identity and activity states with high purity, without the need of intrinsically enforcing independence assumptions. We present as a representative example the case of 14 patterns, where one pattern is of activity type and the rest of identity (Fig. 1). In contrast to ACTIONet, we found that cNMF, cLDA, and Louvain recover identity patterns with lower purity and also tend to assign activity patterns to multiple factors. The latter is particularly pronounced in the cluster-based approach (Louvain), providing support for the relevance of a quantitative state-based approach (as implemented in ACTIONet) over conventional clustering. Among factorization approaches, given its underlying independence assumption, cICA is able to similarly identify all patterns with high purity (Fig. 1c, d). However, unlike AA-based (ACTIONet) decomposition, ICA has the caveat of limited factor interpretability, as the latter does not directly map to actual cell observations, nor is expressed using similar and comparable quantitative values. We provide quantitative support for these observations over multiple realizations of simulated data sets and noise levels (Fig. 1c, Supplementary Fig. 1a).

ACTIONet’s enhanced interpretability stems from the fact that the underlying core decomposition framework (AA) learns dominant patterns defined by a linear mixture of actual samples (cells) in the data set. There are other algorithms that use a similar idea, in particular deterministic column subset selection, which is also similar to the algorithm underlying separable NMF: the successive projection algorithm (SPA). To contrast the ability of ACTIONet to identify representative prototypical cells with that of these two other methods, we performed a comparative analysis of the synthetic data set. In each case, we performed decomposition with an increasing number of hidden factors (*k*) and measured how many of the cell identity patterns have been recovered. Ideally, we expect the total number of covered patterns to increase linearly with the number of hidden factors included. We found that SPA-based methods, both with and without ACTION reduction, are the only methods that can recover all identity patterns when considering *k* = 2, …, 30. However, ACTION reduction enables reaching a saturation point (all existing identity patterns discovered) much faster than SPA alone (Supplementary Fig. 1b–d). This distinction becomes weaker as we progressively increase the noise level of input data. Overall, we verified that the combination of SPA and ACTION decomposition is the only tested approach that is able to discover all patterns at all levels of noise considered. Together, our results demonstrate that ACTIONet is able to recover identity and activity states with superior performance and/or interpretability than existing alternatives.

### Reliable, consistent, and interpretable multiresolution cell-state representation

So far we discussed ACTIONet’s matrix decomposition component as applied to discover a target set of dominant patterns of predefined size (number of components *k*). The practical implementation of the idea of multiresolution cell-state decomposition consists of three steps: (1) applying multiple rounds of decomposition, each time increasing by one the predefined number of components to be discovered, (2) synthesizing decomposition results into a single multilevel lower dimensional representation, and (3) removing outliers and redundant patterns to define a reproducible, parsimonious representation with a priori unknown size. This idea and implementation are fundamentally different to the consensus approach discussed in the section above. A consensus approach is defined using a single, predefined number of patterns. This distinction is important because knowing the most adequate number of existing patterns in real data a priori is not at all trivial. In addition, as shown below, a single resolution level is not sufficient to capture both coarse- and fine-grain patterns. Moreover, and perhaps more importantly, the need for a consensus approach stems from the lack of reproducibility intrinsic to popular decomposition methods. ACTIONet’s multiresolution approach circumvents this latter limitation by providing a systematic way to produce robust results in a single run. For clarity, we refer to the conventional approach of performing decomposition using a fixed, predefined number of components as single-level decomposition and to the concatenated and redundancy-filtered set of factorizations resulting from multiple rounds as multiresolution decomposition.

The independent application of single-level decompositions at increasing levels of resolution inevitably results in high redundancy: the most dominant patterns are recurrently recovered. To systematically reduce such redundancy, and to facilitate interpretation, we developed an algorithm that prunes patterns found at multiple resolutions, identifying as a result a nonredundant, multiresolution set of cell-state patterns. Briefly, we build and cluster a pattern graph to define groups of similar patterns across different levels of resolution into equivalent classes. For each class, we then define a representative pattern by giving priority to the lowest resolution (smallest *k*) at which a pattern of the class was discovered (Methods). Within this framework, the size of each class provides an approximate measure of the pattern dominance. This procedure commonly reduces a multilevel space of ~400 patterns (for *k* = 2, …, 30) to a multiresolution space of ~20 states. For example, we applied this algorithm to the PBMC data and reduced the 464 patterns resulting from factorizations from *k* = 2 to *k* = 30 into a set of 21 dominant multiresolution patterns. Annotation of these dominant patterns enables a fast and intuitive way to interpret the underlying transcriptomic states.

**Fig. 2:**
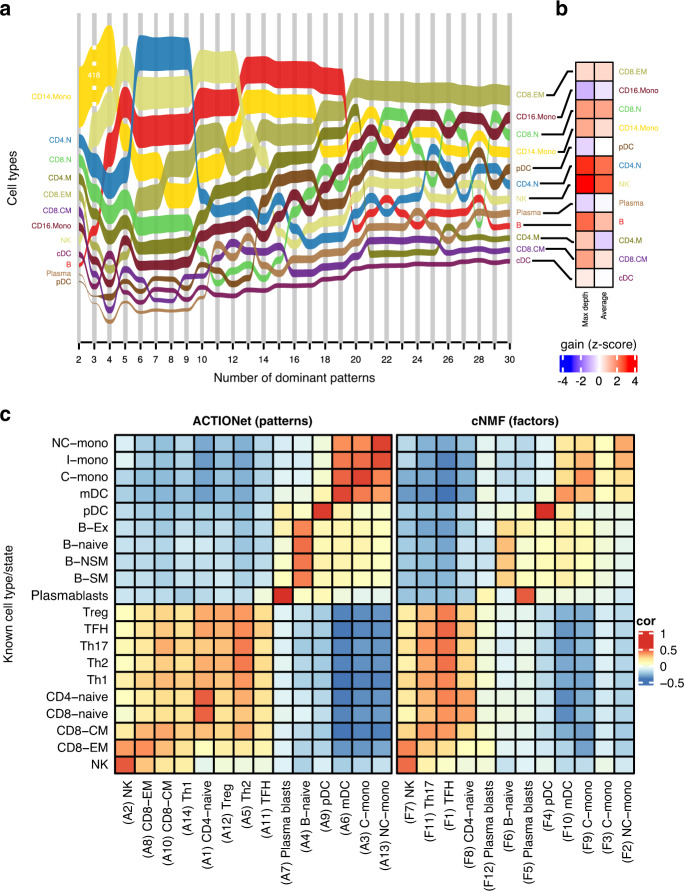
Resolution dependency of cell identity pattern recovery. **a** Performance of ACTIONet decompositions in recovering patterns corresponding to known cell types across increasing resolution levels (number of patterns/archetypes). Lines represent the recovery score of the best-matching cell type. **b** Comparison of cell-type recovery at maximal resolution relative to multiresolution (MR) decomposition (logFold). MR method balances both fine- and coarse-grain patterns, whereas increasing single resolution comes at the price of losing the global coarse-grain pattern of cells with less variability (such as NK cells, here). **c** Interpretability of ACTIONet and cNMF identified patterns (rows) based on their similarity (correlation) with bulk cell-sorted RNAseq profiles for PBMC purified cell-types (columns).

The working principle of ACTIONet is that patterns at different resolutions are complementary, meaningful, and informative. We investigated the ability of multiresolution vs single-level decomposition to recover known cell types. In particular, we asked whether a multiresolution decomposition captures patterns in the data not accessible by means of single-resolution analysis, not even when choosing to predefine a high number of patterns to be recovered. We used human PBMC data, as this system has become a standard in computational single-cell analysis. We considered a data set with a total of 14,311 cells spanning 12 major PBMC cell types^26^. To measure the ability of discovered patterns to recover cell types, we quantified the degree to which the contribution of a given pattern to a cell (encoded in matrix H) is significantly different for cells of a given type relative to the rest. We used these values to define the best-matching pattern for each cell type. This approach allows us to quantitatively trace how well the discovered patterns capture cell-type information as we increase a predefined number of total patterns to be found. We measured such a cell-type capture rate by the log-transformed *p* value of Welch’s paired *t* test. The dynamics of the obtained traces clearly show that capture rates of different cell types are maximized at different levels (Fig. 2a). To provide quantitative analyses, we next used the capture dynamics of single-level decompositions as a reference to compare the capture rates achieved by the multiresolution approach, which we measured independently (Fig. 2b).

To test our intuition that increasing resolution may not always improve cell-type recovery and, therefore, integrating information at multiple resolutions provides a more adequate data representation, we first compared the logfold-change (logFC) in capture rate achieved by multiresolution versus the one obtained at the highest resolution considered (*i* = 30 patterns). Based on saturation analyses we found that increasing resolution beyond this number does not provide additional information (Supplementary Fig. 1f). In support of our intuition, we found a general pattern in which the different cell types are best captured at different resolutions (Fig. 2b). Increasing resolution improves identification of rare or less represented cell types; however, this increase comes with the cost of losing precision for capturing the coarse-grained patterns of more common cell types. Given that it is unknown what resolution will capture best what cell type, a simple averaging across all resolutions would also provide a suboptimal representation. To test this expectation, we compared the logFC of the achieved performance by multiresolution versus the average logFC across resolutions, and we indeed found that the average behavior does not outperform multiresolution in all cases (Fig. 2b). Based on these observations we conclude that multiresolution decomposition provides a balanced representation of cell states in which both fine and coarse-grain patterns are captured without losing power by favoring one or the other. In other words, by considering selected patterns captured at different resolutions ACTIONet is able to learn patterns that recover more closely cell states with different properties, resulting in a useful trade-off between rare and common patterns.

Next, we evaluated the quality and interpretability of multiresolution cell states by comparing the recovered signatures with reference profiles measured previously using bulk RNA sequencing across an extensive panel of cell-sorted populations^27^. To simultaneously address ACTIONet’s ability to recapitulate cell-type expression profiles, and the consistency and particularities of ACTIONet’s estimated profiles with those of alternative methods, we in parallel applied cNMF. We discovered 14 underlying dominant patterns using ACTIONet. In contrast, by following the suggested protocol for a priori selecting an appropriate number of underlying patterns^12^, we recovered 12 pattern profiles using cNMF. We then correlated both pattern profile sets with labeled-RNAseq data. Both approaches recover profiles that are comparable with bulk data; however, ACTIONet was able to recapitulate the expected profiles more precisely. In particular, unlike cNMF, ACTIONet identified a higher correlation among the heterogeneous Myeloid cells (C/NC-Mono and mDC), and identified patterns at a higher resolution such as subtypes of T cells, including CD4 memory cells. The same behavior was not displayed by cNMF, which overall recovered less heterogeneity of cell states and lower consistency with known, matching profiles (Fig. 2c).

### Pattern-based cell network analysis

To build an approximate representation of high-dimensional transcriptional states as lower dimensional shapes or state manifolds^28^, ACTIONet introduces a complex network analysis framework specifically designed to capture the structural representation of the cell-state pattern space. A cell–cell similarity network is built by first integrating the output of all individual decompositions to define a metric cell space, which is then used to construct a compact representation as a sparse network. Unlike conventional graph-based embedding methods, the goal of which is usually to derive an independent structure to use for 2- or 3D layout visualization, and to perform cell clustering^20^; ACTIONet’s network produces a one-to-one correspondence between underlying transcriptomic patterns and the structural representation of the cell-state space. In practical terms, this means that the resulting network topology will directly match the discovered dominant transcriptional patterns and their associations.

The multiresolution cell-state representation facilitates the discovery of a small set of dominant and interpretable cell-state patterns. However, we found that we can profit from the full results of all decompositions at multiple resolutions to identify cell relationships and define a lower dimensional manifold for visualization and exploration. In this context, we empirically found that the redundancy of patterns discovered across resolutions provides more detailed cell associations than when considering one level or only the condensed multiresolution resolution representation. In what follows we show this feature of ACTIONet by means of comparison. We first describe the network building process and then use the PBMC data to demonstrate ACTIONet’s network characteristics, and to contrast with conventional network approaches.

**Fig. 3:**
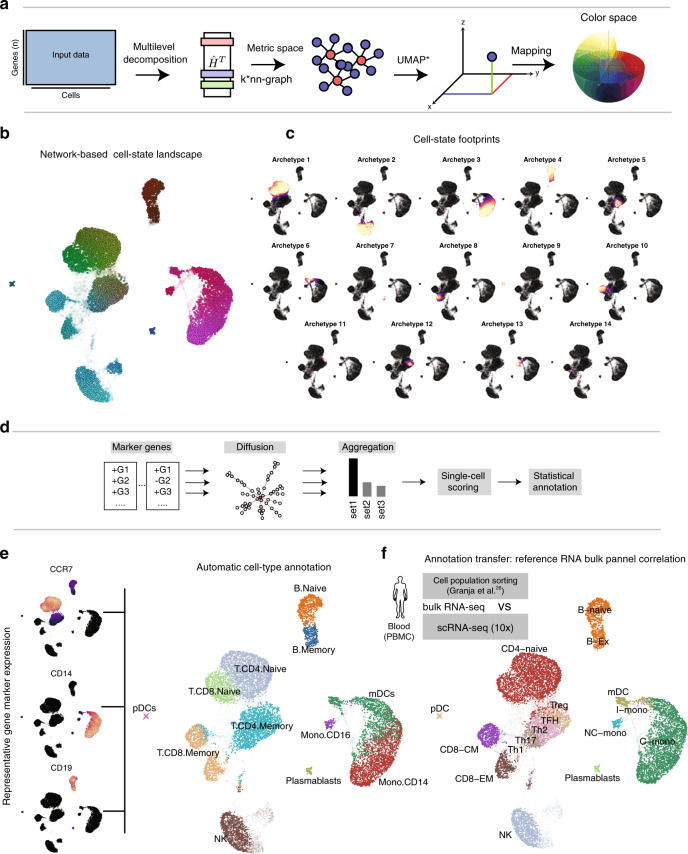
ACTIONet’s network-based analysis. **a** Overview of the network construction process. **b** ACTIONet’s 2D representation of the cell-state landscape. De novo cell coloring captures the underlying heterogeneity of cell space. **c** Multiresolution patterns/archetypes footprint projected on 2D ACTIONet’s network. Footprints capture both fine- and coarse-grain patterns. The majority of identified patterns form cluster-like footprints defining network neighborhoods. **d** Overview of the ACTIONet network-based cell annotation framework. **e** Automated cell-type annotation using known marker genes. **f** Cell annotation inference based on additional data sets—cell-sorted bulk profiles as an example.

ACTIONet’s network building analysis involves four steps: (1) discovery of dominant patterns; performed by independent ACTION decompositions at all resolution considered; (2) definition of a joint cell-state representation; by concatenating the contributions of patterns to cells at all resolutions (defining an encoding matrix H^*^); (3) cell similarity measurement, performed over multilevel profiles using the square root of Jensen-Shannon divergence (JSD) as metric; and (4) network construction; performed using a density-dependent nearest-neighbor algorithm (k* -NN) to connect cells. By using k*-NN, cells are connected based not only on proximity in metric space, but also on the heterogeneity and density of a cell’s neighborhood. This technical improvement avoids the need of defining a fixed number of *k* cell neighbors a priori. The resulting network provides a means to visualize a large-scale state space using efficient graph layout algorithms (Fig. 3a).

Figure 3b shows the network representation of the PBMC transcriptional landscape. To aid intuition, ACTIONet uses by default an automatic coloring scheme (a color space*)* that links transcriptomic with color similarity (Fig. 3ab). Here, cells with similar colors share similar transcriptomic signatures. The network recovers a modular structure, defining cell neighborhoods that usually correspond to cell types and states. ACTIONet’s uses the concept of state pattern footprints to explore how dominant patterns project to the cell network space (Fig. 3c). This analysis explicitly shows how network topology directly corresponds to underlying dominant patterns. Each footprint visually represents the degree to which a given pattern contributes to the transcriptomic state of a cell. Individual patterns tend to explain well the distinct cell network neighborhoods. To facilitate interpretation, it is straightforward to similarly project gene expression patterns of genes relevant to the cellular system in consideration, thereby visually associating neighborhoods (network topology) (Fig. 3b), footprints (pattern activity) (Fig. 3c), and gene activity (marker expression) (Fig. 3e). Using these features, and given that ACTIONet also learns the gene signatures discriminating the patterns, it is possible to automatically infer best estimates of cell annotations, for example, cell-type labels and confidence scores based on sets of marker genes (Fig. 3d). Figure 3e shows ACTIONet’s best estimates of PBMC cell-type labels. Based on this analysis, we confirm that neighborhoods both recover major cell types and locally structure associated cell populations, as supported by marker gene expression patterns. In addition to gene sets, it is possible to define and interpret cell and network neighborhood properties based on external data sources. Cell groups can be associated with independent biological features by correlating the corresponding dominant patterns with external data. To demonstrate this, we used bulk cell-sorted RNAseq profiles and annotated the cell groups based on best-matching profiles Fig. 3f. Thus, ACTIONet provides multiple tools to easily map different sources of evidence to support the interpretation of the transcriptomic patterns underlying the network structure representing the data set under study.

**Fig. 4:**
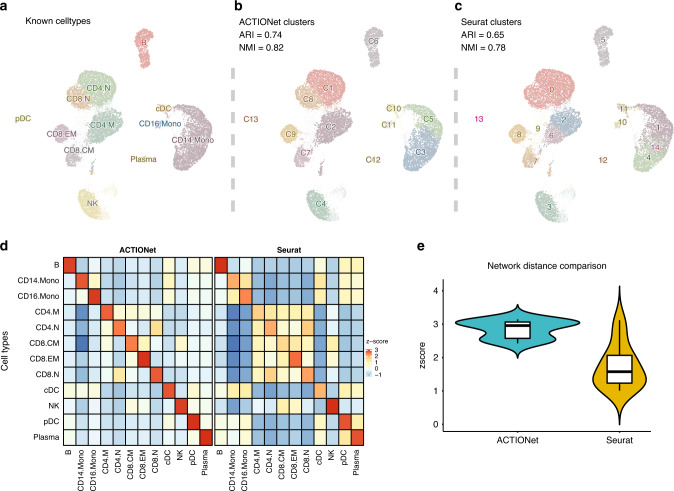
ACTIONet’s framework cluster-based analysis. **a** Cells labeled with known cell type annotations. **b** Performance of ACTIONetʼs network-based clustering in recovering known cell types. **c** Performance of Seuratʼs network-based clustering in recovering known cell types. **d** Heatmap of (aggregate) inter- versus intra-cluster pairwise cell connectivity score. **e** Distribution of intra-cluster pairwise connectivity scores.

Although ACTIONet was not designed for cluster-based analyses in mind, having a high-quality network representation enables the application of a rich set of existing graph algorithms, including clustering. We evaluated the quality of clustering enabled by ACTIONet’s network by measuring known cell-type recovery in the PBMC data, and compared our performance with that of the standard Seurat pipeline (Fig. 4). This analysis demonstrated that, while clustering results are consistent overall, and thus useful for integration with common practices, ACTIONet’s enhanced resolution is able to partition large cell groups into fine clusters leading to a better recovery of known cell types. We quantified cell-type recovery using two membership vector comparison metrics (ARI and NMI) (Fig. 4c, Supplementary Fig. 1g), as well as a quantitative measure of cluster purity (Fig. 4d). The later showed that ACTIONet’s clusters indeed show close agreement with cell types, as evidenced by a near-perfect diagonal pattern in a cluster vs known cell-type annotation purity plot (Fig. 4d). This pattern was not observed when using the alternative method, which instead showed blocks consistent with patterns of grouping and cell-type merging. As ACTIONet’s ability to capture meaningful and reliable cell associations is ultimately encoded in the JSD-based cell similarity measure introduced herein, we tested the degree to which this measure captures the expected high similarity of cells of the same type. Using permutation tests, we demonstrate that ACTIONet’s cell similarity scores are significantly higher for cells of the same type, relative to random expectation. The observed deviation from random expectation is remarkably larger than when considering the distance measure of standard tools (e.g., HVG + PCA + Euclidean, as implemented in Seurat) (Fig. 4e). Taken together, these analyses demonstrate that ACTIONet’s network constructed based on multilevel patterns is able to capture transcriptional signatures with superior resolution.

### Integrative network-based analysis: the cell-state space of the human cortex

**Fig. 5:**
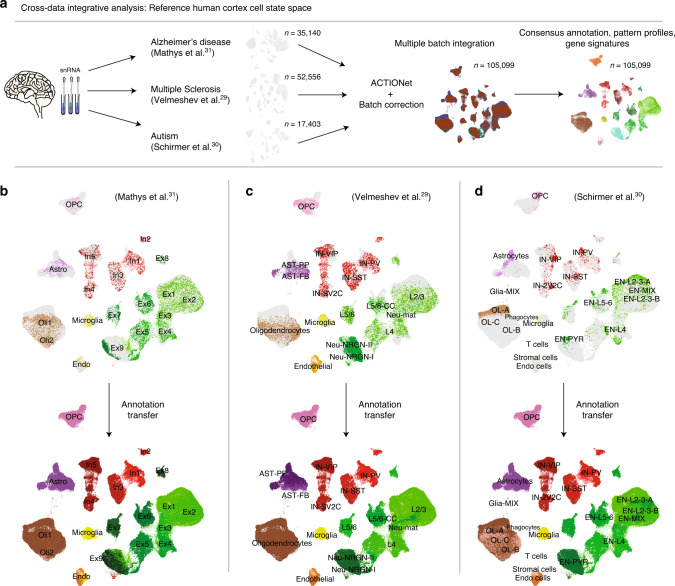
Joint analysis of multiple independent data sets. **a** Overview of integration and batch-correction framework. **b**–**d** Missing label inference using the label-propagation algorithm.

To show how ACTIONet’s pattern-based manifold leaning analysis can be used for cross-data analysis, we performed an integrative analysis of published single-cell transcriptomic studies of the human prefrontal cortex (Fig. 5a). We obtained the control (not pathology related) samples from three recently published studies of brain-related diseases, including Autism spectrum disorder^29^, Multiple sclerosis^30^, and our own study on Alzheimer’s disease^31^. We merged all cells from pathology-free brains as reported in the three studies (*n* = 52,556 cells Velmeshev et al.; *n* = 35,140 Mathys et al.; and *n* = 17,403, Schirmer et al.) into one integrated data set (*n* = 105,099 cells) and directly applied ACTIONet’s pipeline. Similar to all methods for single-cell analysis, AA might be vulnerable to batch effects. Because of this, we have implemented within ACTIONet’s pipeline a kernel reduction function that integrates the recent batch-correction tool Harmony^32^ with ACTIONet reduction step. This approach considering batch-correction kernel reduction circumvents batch-correction limitations, enabling integrative data analysis. Indeed, we observed that cell grouping patterns in the network correspond to cell type and subtype associations, and not to batches, suggesting reliable integration (Fig. 5a).

We use ACTIONet’s integrated output to illustrate two cross-data analysis applications. First, cells from the different studies were originally annotated using different labels, some of which include more detailed and curated subtype annotations. To further demonstrate the power of integration, and how this approach circumvents batch limitations, we use the integrative network to transfer the reported labels of one data set to the rest of cells, effectively inferring annotations for two-thirds of the cells using knowledge from one-third. This approach is based on the application of the guilty-by-association principle across the joint cell space. We show that taking as a reference each data set independently, we can effectively transfer the annotations to define consistent cell neighborhoods corresponding to all known neuronal and glial populations (Fig. 5b–d). Given the consistency of annotation patterns observed across network neighborhoods and data sets, we reasoned that we could use the dominant patterns that explain network structure to define consensus cell annotations and gene signatures.

**Fig. 6:**
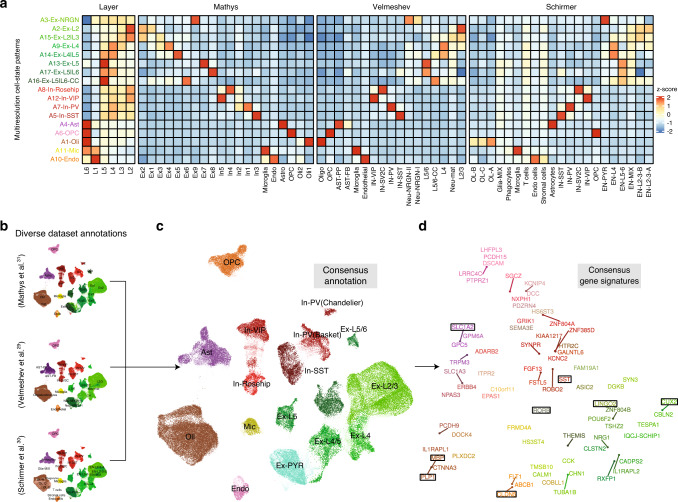
Consensus annotation of human PFC cell types. **a** Overrepresentation analysis of marker sets reported in original studies onto the set of joint patterns identified with ACTIONet across three PFC data sets. **b** Individual cell annotations from each data set and enrichment patterns in **a** are considered to define a consensus cell annotation vocabulary. **c** Consensus annotation based on curated annotations and reference gene set enrichment patterns. **d** Gene view/space of the constructed joint cell space. Top pattern discriminatory genes are plotted in a 2D space with coordinates consistent with ACTIONet’s 2D cell view in **c**.

We first tested whether dominant patterns discovered through multiresolution decomposition recover all reproducible clusters reported in the original studies and are also consistent with corresponding gene markers. For each study and reported cluster, we estimated the degree to which reported marker genes show high discriminatory power for each dominant pattern discovered by ACTIONet (Fig. 6a). By means of resampling-based enrichment analysis, we found that multiresolution cell-state patterns are indeed defined by very specific gene signatures that are reproducible across data sets. To aid interpretation we included reference gene markers of cortical layers, and similarly, we found that patterns recovered signatures of distinct cortical layers. Based on these observations, and the corresponding annotations reported in each study (Fig. 6b), we defined a consensus annotation for each multiresolution cell-state pattern, which we then projected to the cell space (Fig. 6c). Finally, we use ACTIONet to project top pattern discriminating genes onto a consistent 2D space to generate a gene view of cortical transcriptional heterogeneity. This analysis automatically leads to major neuronal (e.g., LINGO2, CUX2, SST) and glial (e.g., SLC1A2, MBP, CLDN5) markers determining the different cell neighborhoods (Fig. 6c).

In summary, ACTIONet’s network-based integrative analysis can be used to homogeneously annotate cells across data sets using a consensus vocabulary and to define a consistent and reproducible set of discriminating gene markers. In the application reported herein, we use such an approach to annotate and define the gene signatures of the currently identifiable transcriptomic cell types and subtypes of the human prefrontal cortex (Fig. 6b, c).

## Discussion

We introduce ACTIONet, a computational analysis framework that introduces and implements the concept of multiresolution cell-state decomposition for application in single-cell transcriptomic analysis. ACTIONet’s working principle is that dominant data-driven patterns at different resolutions are complementary, meaningful, and informative. To make this idea and concept practical, ACTIONet systematically discovers, prunes, and unifies the results of matrix decompositions at different levels of resolution to synthesize a nonredundant, multiresolution set of cell states that capture the complexity of the single-cell transcriptomic profiles. The end product provides a unique way to analyze single-cell states that both solves a technical problem fundamental to matrix decomposition techniques, and that is connected to the intrinsic complex variability captured through single-cell profiling.

**Fig. 7:**
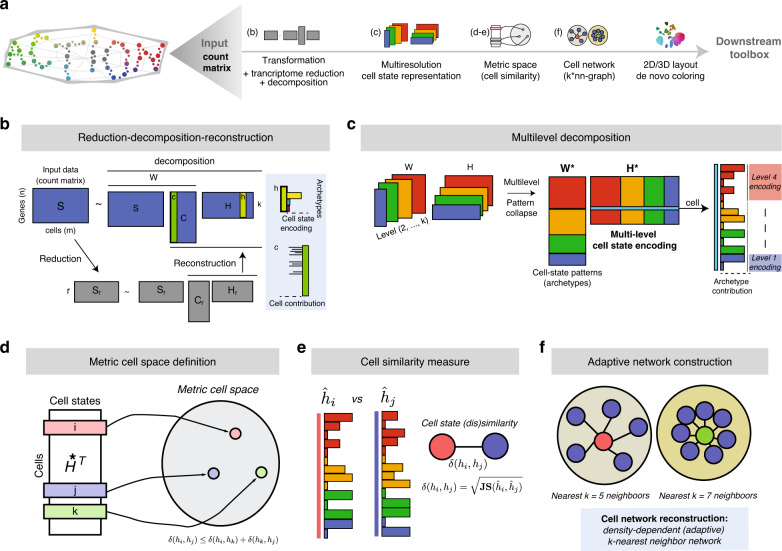
ACTIONet framework overview. **a** Main steps in ACTIONet. **b** ACTION-based matrix decomposition. Dimensionality reduction for feature selection (reduction) is coupled with AA to perform individual-level decomposition and identify *k* latent cell-state patterns (archetypes). A column c of the cell influence matrix C encodes the influence of cells on the patterns. A column h of the cell-state encoding matrix H encodes the relative contribution of each pattern to the transcriptome of each cell. **c** Multilevel ACTION decomposition with an increment in the number of archetypes per level. Concatenation of individual-level matrices defines multilevel encoding (H^*^), cell influence (C^*^), and profile matrices (W^*^). **d**, **e** Metric cell space defined by measuring distances on multilevel cell-state encodings. **f** Construction of a sparse network representation of the cell space.

We implemented this approach, along with a large set of associated downstream analysis tools, in an easy-to-use and freely available computational environment for both R and Python: ACTIONet (https://github.com/shmohammadi86/ACTIONet). In order to make such an approach feasible for large-scale analysis and exploration, ACTIONet includes methodological innovations at multiple steps, each of which could be independently valuable for other applications. ACTIONet uses (1) an ACTION-based implementation of AA for sparse matrix representation, (2) an efficient randomized SVD-based low-rank approximation (reduction step) that enables scaling with input data size, (3) an archetypal-based metric cell space construction that enables measuring cell distances, (4) an adaptive nearest-neighbor algorithm to build a multiresolution network with automatic neighbor size selection, (5) an adaptation of UMAP’s stochastic gradient descent (SGD)-based algorithm to project a multiresolution network into 2 and 3D space, and (6) a network-based feature selection method to robustly identify a nonredundant subset of underlying cell-state patterns. Figure 7 summarizes the technical components of the ACTIONet framework.

Our analyses of diverse data sets demonstrate that the different steps included in ACTIONet individually show superior performance relative to existing alternatives, and that ACTIONet’s framework as a whole can aid the interpretation of data sets of different complexity by straightforward visualization, exploration, and statistical annotation. ACTIONet can contribute to the single-cell community both as an analysis framework to aid biological discovery, as well as technical development to motivate novel methodologies.

## Methods

### Data sources

Synthetic scRNA-seq data sets were obtained from ref. ^12^. The data set contains 20 instances of cells with 13 activity patterns (programs) defining cell types, and one activity pattern affecting multiple cell types. Patterns are repeated at three different levels of noise to measure the robustness of different methods. The PBMC data set used in this study was adopted from a recent profile of 14,311 single cells^26^. The cell-sorted bulk PBMC data set for validation was obtained from ref. ^27^. Data for the joint analysis of the human prefrontal cortex was obtained from three independent studies^29–31^ and includes in total 105,099 cells from control individuals.

### ACTION decomposition

AA^15^ is a matrix decomposition technique that aims to identify a set of underlying exemplary patterns (archetypes) that can optimally represent the identity of the samples. The key identifying feature of the AA is that each archetype is a convex combination of existing samples. As such, archetypal patterns closely resemble actual data points and are highly interpretable. In addition, unlike NMF and ICA, AA does not suffer from rotational ambiguity^17^. In the context of single-cell analysis, each archetype can be seen as a representative cell-state pattern. Formally, AA can be cast as the following optimization problem:

1 $$\begin{array}{l}\min _{{\mathbf{C}},{\mathbf{H}}}\left\| {{\mathbf{S}} - \overbrace {{\mathbf{SC}}}^{{\mathbf{W}}^{(AA)}}{\mathbf{H}}^{(AA)}} \right\|_F^2\\ {\mathrm{Subject}}\,{\mathrm{to:}}\mathop {\sum}\limits_j {c_{:j}} = 1,\mathop {\sum}\limits_j {h_{:j}^{(AA)}} = 1,0 \le c_{ij},h_{ij}^{(AA)}\end{array}$$

in which matrix **S** is the input profile and matrices **W** and **H** are the archetype and cell encoding matrices, respectively. Owing to the convexity of columns in **H**, each data point can be interpreted as a distribution over the archetypes, ${\mathbf{p}}(s_i|w_j)$, and thus is embedded in the simplex spanned by the archetypes. Geometrically, when the number of archetypes is greater than one, they reside on the convex hull of the data^15^. In fact, vertices of the data convex hull are the global minimizer for AA. Therefore, AA can be seen as a low-rank approximation of data convex hull.

Although inherently intuitive and highly interpretable, solving AA is challenging as it can be reduced to an instance of the Euclidean sum-of-square clustering problem, which is known to be NP-hard^33^. As such, all known algorithms can only guarantee local convergence; thus, an efficient and strategic initialization of the optimization procedure can have a drastic effect on both speed and quality of identified archetypes (here cell-state patterns)^34^. To remedy the initialization issue, we first notice that separable non-negative matrix factorization (sepNMF)^35^ can be formulated as a special case of AA^18^:

2 $$\begin{array}{l}\min _{{\cal{K}},{\mathbf{H}}}\left\| {{\mathbf{S}} - \overbrace {{\mathbf{S}}(:,{\cal{K}})}^{{\mathbf{W}}^{(sepNMF)}}{\mathbf{H}}^{(sepNMF)}} \right\|_F^2\\ {\mathrm{Subject}}\,{\mathrm{to:}}\mathop {\sum}\limits_j {h_{:j}^{(sepNMF)}} = 1,0 \le h_{ij}^{(sepNMF)}\end{array}$$

However, unlike AA, NMF can be efficiently solved using the SPA^19,36^, which has theoretical guarantees to find the global optimum solution (under a well-defined noise model). To combine the theoretical guarantees of sepNMF with the flexibility of AA, we first solve the sepNMF problem and then use the inferred factors to initialize **W**^(*AA*)^ for the AA inference problem. This process ensures the reproducibility of results and considerably enhances convergence properties. Intuitively, AA takes as input a set of pure (prototype) cells identified using sepNMF and locally adjusts these prototypes by shifting and sparsely averaging in the proximal neighborhood of the identified solutions. The enforced convexity (parsimony assumption) on the columns of **C** ensures sparsity, resulting in the definition of every archetype as a local average of a small number of cells. This approach balances the need for averaging, reduces noise, and imputes missing values while preserving subtle transitions in distinct cell states.

### Reduced kernel-based ACTION decomposition

One key assumption in AA is the linear dependencies between data points and archetypes, which is enforced by the convexity of matrices **C** and **H**. However, the original data space might not be optimal to linearly represent archetypes. To address this issue, we first notice that AA is amenable to the kernel trick^17^:

3 $$\min _{{\mathbf{C}},{\mathbf{H}}}\left\| {{\mathbf{S}} - {\mathbf{SCH}}} \right\|_F^2 = \min _{{\mathbf{C}},{\mathbf{H}}}{\mathbf{tr}}(\underbrace {{\mathbf{S}}^T{\mathbf{S}}}_{\mathbf{K}} - 2\underbrace {{\mathbf{S}}^T{\mathbf{S}}}_{\mathbf{K}}{\mathbf{CH}} + {\mathbf{H}}^T{\mathbf{C}}^T\underbrace {{\mathbf{S}}^T{\mathbf{S}}}_{\mathbf{K}}{\mathbf{CH}})$$

Mohammadi et al.^18^ introduced a gene expression kernel by applying a series of biologically-motivated transformations to reduce the effect of highly expressed but uninformative genes while simultaneously boosting the contributions of highly specific genes. This approach automatically reweights genes in each cell and does not rely on the preselection of highly variable genes (HVGs). However, constructing the kernel matrix for large data sets hinders the scalability of ACTION. To remedy this issue, we note that ACTION kernel can be explicitly written as **K**_*ACTION*_ = **Z**^*T*^**Z**, where **Z** is the transformed expression matrix. Using this notation, one can use a rank-D approximation of the kernel matrix instead. In the case of ACTION kernel, this can be accomplished by using the reduced SVD of the matrix **Z**, denoted by *S*_*r*_, as the input to the ACTION decomposition. To further enhance scalability, we implemented a fast randomized implementation of the SVD algorithm that has been recently developed to take advantage of the sparsity structure of the input matrix^37^. For general kernels, the Nyström method^38^ is used as an efficient technique for the eigenvalue decomposition of the kernel matrix.

### Multilevel decomposition and hierarchical cell similarity

Having to preselect and use only an optimal value of *k* for decomposition is a fundamental limitation of most common matrix decomposition techniques (including ACTION). Our observations of real single-cell data suggest that different values of *k* have different power and resolution to identify coarse vs fine-grained patterns of variability. In ACTIONet we provide a practical solution to this problem. Our solution enables gathering information across different decomposition levels. We independently perform ACTION-based decompositions, while gradually increasing the number of *k* archetypes. We then concatenate a posteriori all archetype profile matrices (**W**) and all cell encoding matrices (**H**) to define a multilevel cell-state (archetypal) profile W^*^ and corresponding multilevel cell-state encoding matrix H^*^. We then use these two structures to reconstruct and discover multiresolution dominant patterns.

We use the multilevel profiles encoded in matrix H^*^ as low-dimensional quantitative representations of the state of single cells. The set of encodings thus jointly defines an observable cell-state space. To make the theoretical construct of a cell-state space of practical use, we defined a metric space over cells using cell encodings. Importantly, this metric space must respect the geometric requirements of the triangle inequality. We first rescale each column of H^*^ to have sum 1 and treat it as a distribution of cells in the state space of archetypes. Next, we use the normalized H^*^ to construct a metric space capturing complex relationships among cells. Let us denote with *h*_*i*_ and *h*_*j*_ two arbitrary columns of the normalized H^*^ matrix.

The Kullback–Leibler (KL) divergence between *h*_*i*_ and *h*_*j*_ is defined as:

4 $${\mathbf{KL}}(h_i\parallel h_j) = - \mathop {\sum}\limits_x {h_i} (x){\mathbf{log}}\left(\frac{{h_j(x)}}{{h_i(x)}}\right)$$

which is a positive but asymmetric measure of the difference between distributions *h*_*i*_ and *h*_*j*_. We then use KL divergence to define the symmetric Jensen-Shannon divergence as follows:

5 $${\mathbf{JS}}(h_i\parallel h_j) = \frac{1}{2}\{ {\mathbf{KL}}(h_i\parallel m) + {\mathbf{KL}}(h_j\parallel m)\}$$

In which $m = \frac{1}{2}(h_i + h_j)$. This measure is both symmetric and bounded between [0, 1], given that base 2 is used for computing the logarithm^39^. Finally, we note that JS divergence is not a metric since it does not satisfy the triangle inequality. However, it has been shown that a mono-parametric family of metrics can derive from the JS divergence. In particular, the square root of the JS divergence satisfies all requirements of a metric^40^. Thus, we compute the pairwise distance between single-cell transcriptomes encoded in the corresponding columns of normalized H^*^, as follows:

6 $$\delta _{ij} = \sqrt {{\mathbf{JS}}(h_i\parallel h_j)}$$

### Fast density-dependent multilevel network reconstruction

The complete cell metric space defined above provides a comprehensive specification of the observable cell-state space. However, it is usually infeasible to store and analyze the complete distance matrix. In ACTIONet we circumvent this problem by constructing a sparse structural representation matching the metric space in the form of a graph. We use an approximate nearest-neighbor search approach based on the hierarchical navigable small world graphs to speed-up network construction^41^. To avoid over- and/or under-fitting to cell types with low/high transcriptional heterogeneity, we extend conventional nearest-neighbor algorithms by employing a local density-adaptive strategy. We adopted the k^*^-nearest neighbors algorithm^42^ originally developed for network-based regression/classification tasks, and modified it to fit our unsupervised network construction task. k^*^-NN algorithm chooses the optimal number of neighbors for each node individually, based on the distribution of neighbors’ distances. This results in a density-dependent nearest-neighbor graph. Formally, for each vertex (cell) *i*, we identify the optimal number of the neighbors to choose using the following procedure:

$$\begin{array}{l}\beta = {\mathbf{sort}}(\kappa \times \delta _{i:})\\ {\mathrm{{\Sigma} }}_\beta = {\mathrm{{\Sigma} }}_\beta ^2 = 0\\ \lambda _1 = \beta _1 + 1\\ {\mathbf{for}}(n = 1 \cdots (N - 1))\\ \quad {\mathbf{if}}(\lambda _n \le \beta _n)\\ \quad\quad n_{opt} = n\\ \quad\quad {\mathbf{break}}\\ \quad {\mathrm{{\Sigma} }}_\beta = {\mathrm{{\Sigma} }}_\beta + \beta _n\\ \quad {\mathrm{{\Sigma} }}_\beta ^2 = {\mathrm{{\Sigma} }}_\beta ^2 + \beta _n^2\\ \quad \lambda _{n + 1} = \frac{1}{n}({\mathrm{{\Sigma} }}_\beta + \sqrt {n + ({\mathrm{{\Sigma} }}_\beta )^2 - n{\mathrm{{\Sigma} }}_\beta ^2} )\\ {\mathbf{return}}(n_{opt})\end{array}$$

where *κ* parameter adjusts the overall desired sparsity of the constructed network. Larger values of *κ* result in more sparse representations, whereas smaller values of *κ* result in more dense networks. In all our analyses we use *κ* = 1, that is, we use the original distances without rescaling.

### Multiresolution cell-state pattern identification

Multilevel decomposition provides a comprehensive list of archetypes, each of which acts as a proxy for a potential cell-state. However, two issues remain to be addressed: (i) when the resolution is low (having a small number of total archetypes), some of the identified cell states are too generic, for which they represent multiple distinct cell states that are well-represented and separate at higher resolution, and (ii) same cell states can be identified in multiple levels of resolution. The latter issue is particularly challenging because it introduces multicollinearity in the resulting archetype matrix profile. It is also of significance as different cell types are best captured in different levels of resolution. We, therefore, developed an approach to remove redundancy, while keeping the most dominant patterns. ACTION decomposition learns a set of influential cells encoded in the matrix **C**. That is, each dominant transcriptional pattern is defined in terms of a linear mixture of cells for which the algorithm learns nonzero coefficients in the corresponding column of **C**. We have empirically observed that the size of the influential cell set is independent of data set size. This unique property of AA-based decomposition allows the identification of a discrete group of cells underlying each pattern. We exploit this property to define nonredundant patterns across decomposition resolutions, and to define their corresponding matrices **C** and **H**. To this end, we define a patterns graph, where each node represents a pattern, and the degree of overlap of influential cells defines the weights of the links connecting them. The degree of similarity for each pair of patterns is quantified by estimating the tail probability of overlap between the set of the observed influential cells. We then applied the Leiden graph-clustering algorithms over this pattern graph to define equivalent patterns classes (patterns clusters). Finally, for each equivalent class, we keep the pattern discovered at a lower resolution, assuming this pattern already captures the corresponding information by means of the encoding of a similar set of influential cells. The corresponding columns and rows extracted from profile (W^*^) and cell-state encoding H^*^ matrices will then define the multiresolution cell-state decomposition.

### ACTIONet network layout

ACTIONet provides a powerful computational tool amenable to well-established network analysis techniques. For efficient graph visualization, we implement low-dimensional graph embedding in 2- and 3D, while preserving the underlying manifold structure. We partially adopted, slightly modified, and reimplemented the stochastic gradient approach used in the Uniform Manifold Approximation and Projection (UMAP), which preserves both global and local topological features in the reduced space^43,44^. We adopted the embedding stage of the UMAP algorithm to layout the density-dependent multilevel ACTIONet graph.

Intuitively, UMAP efficiently approximates a force-directed layout algorithm. At its core, it aims to minimize the cross-entropy of two fuzzy sets (1-simplices), one of which captures relationships between cells in the original graph and the other within the lower dimensional Euclidean subspace. The cross-entropy objective can be written as the sum of an entropy term within the projected subspace, coupled with a KL divergence term that penalizes for the deviation of point distribution from the original edge weights. These components effectively define the repulsive and attractive forces of the layout algorithm. ACTIONet implements the SGD algorithm of UMAP to efficiently optimize the following objective function:

7 $$\mathop {\sum}\limits_{i,j} {w_{ij}} log\frac{{w_{ij}}}{{f(d_{ij})}} + (1 - w_{ij})log\frac{{1 - w_{ij}}}{{f(1 - d_{ij})}}$$

where *w*_*ij*_ is affinity scores corresponding to the edge weights of ACTIONet graph, *d*_*ij*_ are the euclidean distances between embedded points, and the function *f*() is defined as:

8 $$f(d_{ij}) = \frac{1}{{1 + ad_{ij}^{2b}}}.$$

In this formulation, parameters *a* and *b* are inferred through nonlinear least-squares fitting and can be used to control the compactness of the final embedding. By testing a wide range of values, we found that these parameters behave similarly in particular regimes. To simplify the interaction with the algorithm, we precomputed a set of *a* and *b* parameters and replaced them with a new “compactness” parameter, which takes values between 0 and 100 and provides a practically useful range of gradually increasing compact representations for single-cell data sets.

Finally, we observed that UMAP performance improves by using smoothed weights computed as follows:

9 $$w_{ij}^{(smoothed)} = e^{ \,- \,\frac{{\delta _{ij} - \rho _i}}{{\sigma _i}}}$$

where *ρ*_*i*_ is the distance to the closest neighbor of node *i*, and *σ*_*i*_ is set to be the value such that:

10 $$\mathop {\sum}\limits_{i = 1}^k {e^{ \,-\, \frac{{\delta _{ij} - \rho _i}}{{\sigma _i}}}} = log_2(k)$$

where *k* is the number of the nearest neighbors of the node *i*. We use this latter procedure in ACTIONet to convert metric distances between cells into edge weights.

### De novo coloring

We observed that a three-dimensional embedded space usually provides a very good approximation to the apparent structure of the cell-state space, a feature that is sometimes misleadingly lost in the more standard 2D visualization. To directly link the two representations, whereas highlighting the quantitative nature of the cell-state characterization enabled by ACTIONet, we introduce an automatic (de novo) coloring scheme. We first construct both 2D and 3D embeddings and use properly scaled 3D coordinates to map each cell to a color space. We adopt the CIELAB color space which, unlike RGB space, is perceptually uniform: i.e, it is designed such that points with similar distances are visually perceived as having similar color differences^45^. This procedure allows us to intuitively map the three-dimensional embedding of the ACTIONet graph into a color space that recapitulates its overall topology. By using these colors in multiple visualizations, ACTIONet adds information about the quantitative state of cells while linking 2-, 3D cell, and gene views.

### ACTIONet automatic cell annotation

ACTONet’s cell annotation tool uses known marker genes, both positive and negative markers, to infer the most likely cell type for each cell, individually. First, it uses a network-based diffusion method over the ACTIONet network to impute the expression of every marker gene. Then, it computes the signed average of the imputed expression vectors to compute a score for each cell type/cell pair. Finally, it assesses the significance of these cell type-association scores by a permutation test, sampling the same number of imputed genes selected at random, and constructs a null model for their corresponding weighted average statistics. Finally, it uses this null model to assign a *z* score to each cell-type/cell association.

### Network propagation-based missing label inference

We use a variant of the label-propagation algorithm to update a given set of labels using the topology of the ACTIONet network. In brief, we aggregate labels within the neighborhood of each cell and identify the most likely label for each cell, together with a confidence score. If the neighborhood-inferred label for a cell is different from its previous label, and if the ratio of confidence scores is above a threshold, we switch the old labels to the newly inferred label. We perform this operation synchronously for all cells and iterate for a fixed number of iterations or until convergence. As pointed out in previous work^46^, these naive approaches have the drawback of being influenced by the most dominant label in the network. To account for this situation, we built a random model that assesses the total number of observed labels for each class within the neighborhood of cells, relative to their null distribution across the whole graph, as well as edge weights connecting cells to their neighbors. We use these computed *p* value bounds in our framework instead of empirical label frequencies.

### Automatic cell-state annotation

Given a continuous measurement at the cell level, we infer its enrichment at the cell-state pattern level using a permutation test. More specifically, we assess the overrepresentation of cell measurements with the cell-state encoding. For qualitative measurements, such as cell types, we construct its one-hot-encoding and similarly perform a permutation test.

### Patterns saturation analyses

Maximum values for single-level decomposition resolution we investigated through empirical saturation analyses. The sparsity level (number of nonzero elements) of columns in C was recorded as *k* increases. Line plots of this behavior showed that pattern recovery smoothly converges to a point where trivial patterns (columns with only a single nonzero element in C) start appearing. This behavior stems from the ACTION decomposition framework. In brief, in the ACTION framework, a solution is initialized with the output of separable NMF, which provides exactly one nonzero element per column. In the further refinement phase based on AA analysis, if the total number of patterns to be found exceeds what is needed to explain the variability observed in the data, the remaining columns remain unchanged. The reason for this is the sparsity constraint enforced on columns of C, which penalizes the total number of nonzeros in each column. Saturation analyses show that the number of nontrivial columns (with more than one nonzero element) increases as the total number of patterns to be found is increased, until it reaches the point that the number of cell types is less or equal to the number of patterns, in which case the number of nontrivial columns plateaus (Supplementary Fig. 1). Convergence is usually reached with values *k* < 30.

### Reporting summary

Further information on research design is available in the Nature Research Reporting Summary linked to this article.

## Supplementary information

Supplementary Information

Reporting Summary

## Data Availability

Data for synthetic analysis have been obtained from ref. ^12^ via 10.24433/CO.9044782e-cb96-4733-8a4f-bf42c21399e6. The human PBMC data set is from ref. ^26^ and downloaded from https://github.com/GreenleafLab/MPAL-Single-Cell-2019. Data sets related to the human brain (PFC) have been obtained from three separate studies^29–31^.

## Code availability

ACTIONet implementation in C++ with interfaces for R and Python is available from: https://github.com/shmohammadi86/ACTIONet. An automatically generated docker image of the latest release is available from: https://hub.docker.com/r/shmohammadi86/actionet.
