# Supplementary material for: A multiresolution framework to characterize single-cell state landscapes
Source: Nat Commun. 2020 Oct 26;11:5399. doi: 10.1038/s41467-020-18416-6 (PMC7588427; doi:10.1038/s41467-020-18416-6)
Supplement: Supplementary file 1 — Supplementary Information [file 41467_2020_18416_MOESM1_ESM.pdf]

Supplementary Information

A multiresolution framework to characterize single-cell state landscapes

Mohammadi et al.

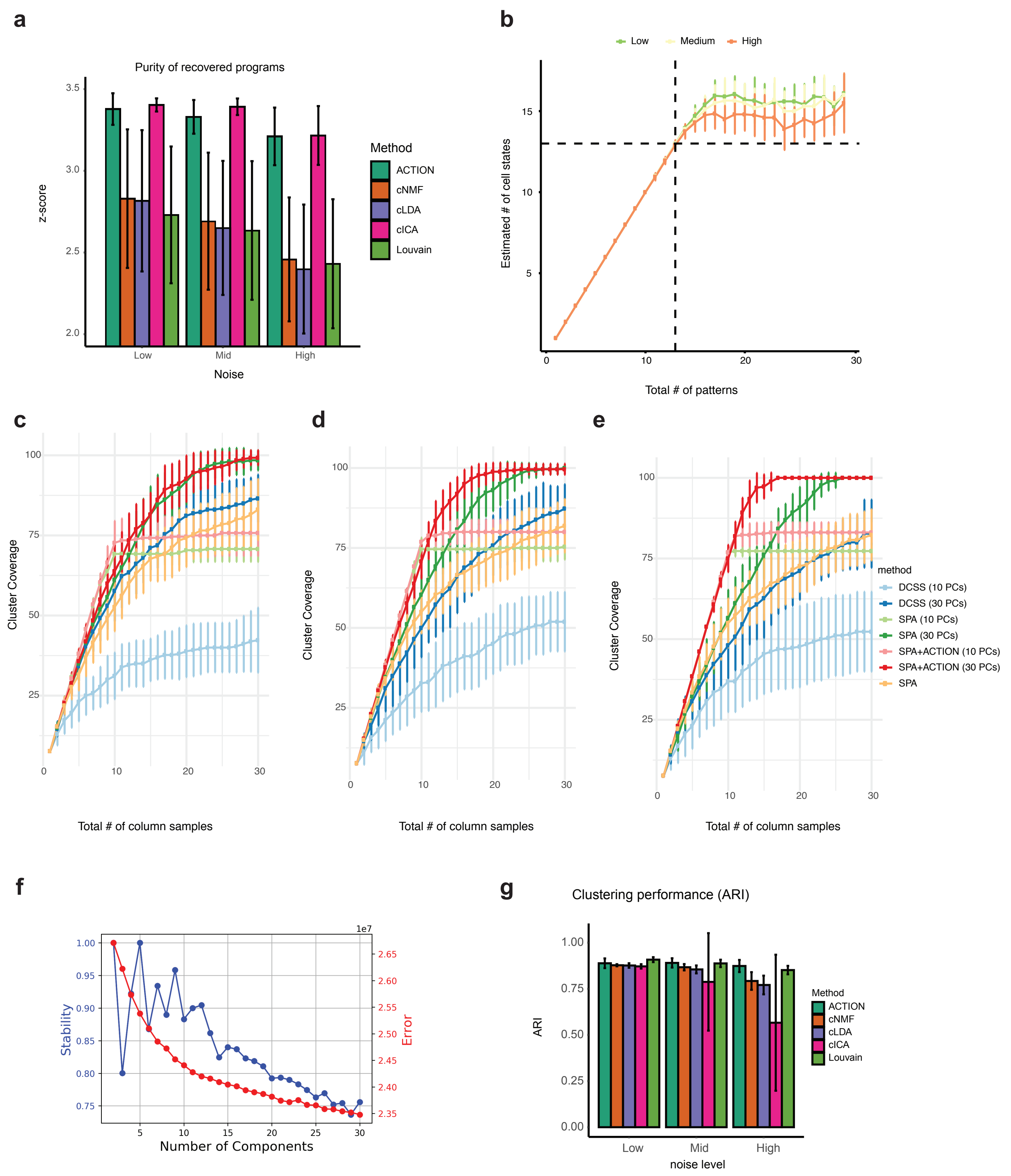

Supplementary Figure 1.
(a) Performance measure of different matrix decomposition methods to recover known underlying patterns. A z-score was calculated as a measure of pattern purity. Performance was assessed across different levels of added noise. n=20 independent samples over each of n=3 independen noise levels. Data are presented as mean values +/- SD. (b) The dynamics of the total number of nontrivial identified patterns as a function of total number of patterns. The total number of patterns exceeds the true number of identity patterns, and number of nontrivial patterns plateaus, suggest ing an empirical approach to identify an appropriate number of underlying patterns. (c-e) Comparative performance of separable NMF (computed using SPA algorithm), with and without ACTION reduction, versus deterministic column-subset selection (DCSC) algorithm. In all cases SPA+reduction outperforms other methods in sampling a more diverse set of underlying patterns. (f) Stability plot of cNMF used to empirically choose an appropriate number of factors. (g) Alternative measure of clustering quality to evaluate the performance of different methods on synthetic data. n=20 independent samples over each of n=3 independen noise levels. Data are presented as mean values +/- SD.
